# A new assay for the simultaneous identification and differentiation of *Klebsiella oxytoca* strains

**DOI:** 10.1007/s00253-016-7881-1

**Published:** 2016-10-08

**Authors:** Karolina Stojowska-Swędrzyńska, Beata Krawczyk

**Affiliations:** Department of Molecular Biotechnology and Microbiology, Gdańsk University of Technology, ul. G. Narutowicza 11/12, 80-233 Gdańsk, Poland

**Keywords:** *Klebsiella* spp., *Klebsiella oxytoca*, *Enterobacteriaceae*, *peh*X gene, PCR, LM PCR typing methods

## Abstract

*Klebsiella oxytoca* is the second most frequently identified species of *Klebsiella* isolated from hospitalized patients. *Klebsiella* spp. is difficult to identify using conventional methods and is often misclassified in clinical microbiology laboratories. *K. oxytoca* is responsible for an increasing number of multi-resistant infections in hospitals because of insufficient detection and identification. In this study, we propose a new simple method called *pehX*-LM PCR/XbaI, which simultaneously indicates *K. oxytoca* species and genotype by the fingerprint pattern. The *pehX*-LM PCR/XbaI is a combination of the following two methods: species-specific amplification of *pehX* gene and non-specific amplification of short restriction fragments by the LM PCR method. The specificity and the discrimination power of the *pehX*-LM PCR/XbaI method were determined by typing 209 *K. oxytoca* strains (included 9 reference strains), 28 *K. pneumoniae*, and other 25 strains belonging to the *Enterobacteriaceae*. The typing results were confirmed by the PCR melting profile method. Unlike the known fingerprinting methods, the *pehX*-LM PCR/XbaI leads to a clear pattern (approx. 3–5 bands) with a sufficient, relatively high discriminatory power. As a result, the time and cost of a single analysis are lower. The method can be used both in clinical and environmental research.

**Electronic supplementary material:**

The online version of this article (doi:10.1007/s00253-016-7881-1) contains supplementary material, which is available to authorized users.

## Introduction

According to the Orskov (1984) taxonomic classification, the *Klebsiella* genus includes *Klebsiella pneumoniae* subsp. *pneumoniae*, *K. pneumoniae* subsp. *ozaenae*, *K. pneumoniae* subsp. *rhinoscleromatis*, *Klebsiella oxytoca*, *Klebsiella terrigena*, *Klebsiella planticola*, and *Klebsiella ornithinolytica*. *Klebsiella* spp. belong to clinical and environmental species/subspecies with a varying degree of virulence. *K. pneumoniae* and *K. oxytoca* are the most commonly isolated *Klebsiella* species in hospital infections (Asensio et al. 2000). These opportunistic human pathogens are involved in serious infections such as pneumonia, sepsis, neonatal septicemia, wound infections and urinary tract infections (Biehle et al. 2015; Brisse and Verhoef 2001). *K. pneumoniae* is the most common cause of hospital respiratory infections in premature neonates in Intensive Care Units and the second most common cause of urinary tract infections and bacteraemia (Giamarellou et al. 2013; Girometti et al. 2014; Han et al. 2015). Whereas, *K. oxytoca* can also be cultured from skin, mucous membranes, oropharynx, intestines and other tissues in healthy humans and animals. It is thought to be an opportunistic pathogen responsible for nosocomial infections in hospitalized patients and a causative organism for antibiotic-associated hemorrhagic colitis (AAHC) in humans. *K. oxytoca* may also be especially pathogenic due to the secretion of cytotoxin (Darby et al. 2014). *Klebsiella* spp. are difficult to identify by conventional methods and often misclassified in clinical microbiology laboratories because they share a similar biochemical profile with several other species (Martínez et al. 2004; Monnet et al. 1991; Monnet and Freney 1994; Podschun and Ullmann 1998; Rosenblueth et al. 2004; Westbrook et al. 2000).

*K. oxytoca* can be detected using a combination of the API 20E test (bioMérieux, France) and other molecular tests for a complete identification. Several studies used the sequencing analysis of 16S rRNA (Lopes et al. 2007), 16S-23S rDNA intergenic spacer region (García-Martínez et al. 1999) and housekeeping genes including the *rpoB* gene (RNA polymerase β-subunit), *nifH* (nitrogenase reductase), *gyrA* (DNA gyrase A subunit), *phoE* (phosphoporine E), *mdh* (malate dehydrogenase), *infB* (translation initiation factor 2), *pehX* (polygalacturonase) (Kovtunovych et al. 2003; Rosenblueth et al. 2004) to identify bacterial species and subspecies. *K. oxytoca* is responsible for an increasing number of multi-resistant infections in hospitals; therefore, epidemiology studies are essential to the understanding of the transmission dynamics in infections caused by these bacteria. It is of utmost importance to determine the degree of relatedness with the use of the genotyping methods. The clonality and phylogenetic diversity of these strains has been established by 16S rRNA gene, *rpoB*, *gyrA*, *gapDH*, and *blaOXY* sequencing (Fevre et al. 2005) and by molecular fingerprinting methods such as Pulsed Field Gel Electrophoresis (PFGE) (Izdebski et al. 2015), Multi Locus Sequences Typing (MLST) (Izdebski et al. 2015), Internal Transcribed Spacer Polymerase Chain Reaction (ITS PCR) (Stojowska et al. 2009a, b), Amplified Fragment Length Polymorphism (AFLP) (Jonas et al. 2004), Randomly Amplified Polymorphic DNA (RAPD) analyses (Brisse and Verhoef 2001) and PCR Melting Profile (PCR MP) (Stojowska et al. 2009a, b). Although PFGE is considered to be the gold standard for the detection of clonality in outbreaks, PCR-based methods are cheaper and provide faster results. The AFLP technique can be used to determine the inter- and intraspecies relatedness; however, as the use of it requires a high level of technical competence it is not recommended for routine bacteria identification. The ITS PCR method has a low level of discriminatory power and was chosen to study the genetic similarities and relationships between *K. oxytoca* strains isolated over long periods of time and across large geographical ranges (Stojowska et al. 2009b). The RAPD analysis is faster and technically less demanding but for the results to be reproducible the PCR conditions need to be carefully standardized (the choice of a primer, DNA and primer concentrations, polymerase type or a thermal cycler profile are critical to producing informative patterns) (Brise and Verhoef 2001). Another method, the PCR MP is sensitive to temperature fluctuations and for this reason, to generate reliable and repeatable data the thermal cycler needs to be calibrated regularly (Krawczyk et al. 2006; Stojowska et al. 2009a). If patterns are too complex, fast typing is difficult to achieve, especially in hospital settings. In addition, fingerprint patterns do not provide species- or genus-specific information regarding the tested strains. The purified DNA from correctly identified species of bacteria needs to be used. Closely related species of the same genus may make the correct diagnosis difficult.

In this study, we propose a new one-step assay for the simultaneous species identification/confirmation and strain genotyping with the use of a PCR reaction.

## Materials and methods

### Bacterial strains

The *K. oxytoca* we used for the purposes of the study were provided to us by the State Institute of Hygiene (Poland). We studied nine reference *K. oxytoca* K serological type from Ørskov’s collection (K26, K29, K32, K41, K44, K65, K66, K68 and K72) (Ørskov and Ørskov 1984) and more than 200 *K. oxytoca* strains isolated from patients hospitalized in various clinical facilities across Poland between 1954 and 2007 (14 of them were isolated in 1999 from patients of Neonatal Intensive Care Unit of a single hospital in Bydgoszcz and were marked as closed related strains from nosocomial infection). All the strains were previously classified as *Klebsiella oxytoca* by the Department of Bacteriology at the National Institute of Public Health (Poland) and tested for genetic diversity in 2009 (Stojowska et al. 2009a, b). We used *K. pneumoniae* subsp. *ozaenae* (ATCC 25926), *K. pneumoniae* subsp. *pneumoniae* (ATCC 700603™) and *K. pneumoniae* subsp. *rhinoscleromatis* (ATCC® 13884™) as reference strains, and 25 clinical *K. pneumoniae* which are not *Klebsiella oxytoca.* Furthermore, we tested 25 clinical strains of other *Enterobacteriaceae* species (10 strains of *Escherichia coli*, 10 strains of *Serratia marcescens* and 5 strains of *Proteus vulgaris*) from the Molecular Biotechnology and Microbiology Department of the Gdańsk University of Technology. All the clinical isolates were identified biochemically using the Mini API ID 32E systems (bioMèrieux, France). *K. pneumoniae* clinical strains were put to the indole test (negative result), the growth test at 10 °C (negative result), and were tested for the production of gas from lactose at 44.5 °C (positive reaction).

### DNA isolation

DNA isolations (from a single colony on an agar plate) were carried out with the Genomic DNA Kit (Bioline, A Meridian Life Science Company). The DNA concentrations were measured using NanoDrop ND-100 (Thermo Fisher Scientific, Wilmington, USA) and were at a level of 10–15 ng per microlitre.

### Species-specific sequence amplification (pehX-PCR)

We have amplified a 344 bp fragment of the pehX gene by PCR according to the Kovtunovych et al. (2003). We used *pehX-*PCR on 25-μl samples using 10 pmol of each *pehX*-specific primer (PEH-A: 5′-GGACTACGCCGTCTATCGTCAAG-3′ and PEH-D: 5′ TAGCCTTTATCAAGCGGATACTGG 3′), 2.5 μl of a deoxynucleoside triphosphate mixture (a 2 mM concentration of each), 1 U of *Taq* polymerase (1 U/μl, Fermentas UAB, Vilnius, Lithuania), 2.5 μl of 10× PCR buffer *Taq* with (NH_4_)_2_SO_4_ (Fermentas UAB, Vilnius, Lithuania), 750 mM Tris-HCl pH 8.8 at 25 °C, 200 mM (NH_4_)_2_SO_4_, 0.1 % (*v*/*v*) Tween 20), 2 μl of 20 mM MgCl_2_, and 1 ng of the genomic DNA. The PCR conditions were as follows: 5 min of initial denaturation at 94 °C followed by 25 cycles of 30 s at 94 °C, 30 s of annealing at 60 °C with extension step at 72 °C for 30 s. After the final cycle, the samples were incubated for 5 min at 72 °C. The PCR products were confirmed for all *K. oxytoca* strains using agarose gel electrophoresis. The *peh*X-PCR products were sequenced for several reactions to ensure that the correct DNA target was amplified (Genomed, Poland). Following this, the sequences were compared with *peh*X gene sequences present in the GenBank database (access number AY065648). For the purposes of the assay, we designed a new reverse primer—pehX8: 5′-CACCGTAAAGGCATACTCCGTATC-3′ which was different from PEH-D that used by Kovtunovych et al., (2003) whilst the forward primer was the same as PEH-A and we called it pehX1. The reaction profile of *pehX*-PCR was performed as described above. The PCR product had a length of 193 bp. The specificity of PCR assay was examined for 209 strains of *K. oxytoca* and 28 *K. pneumoniae*.

### Adapter preparation

The double-stranded adapter (5′ adXbaI) was formed by mixing 20 pmol of each adapter oligonucleotide (aXbaLIG 5′-CTCACTCTCACCAACGTCGAC-3′; and aXbaHELP 5′-CTAGGTCGACGTTGG-3′) with water to bring the sample volume to 50 μl. The mixture was then heated up to 70 °C for 2 min and, following this, slowly cooled down to room temperature, with the result that the adapter was formed at a final concentration of 1 pmol/μl.

### LM PCR/XbaI template preparation

Twenty-five nanograms of genomic DNA was digested using 5 U of the restriction enzyme *Xba*I (10 U/μl, Fermentas UAB, Vilnius, Lithuania) in 20 μl reaction volumes containing a 1× Y/Tango restriction buffer (3.3 mM Tris-CH_3_COOH (pH 7.9 at 37 °C), 1 mM (CH_3_COO)_2_Mg, 6.6 mM CH_3_COOK, 0.1 mg/ml BSA; Fermentas UAB, Vilnius, Lithuania) for 30 min at 37 °C. To achieve ligation, we used a master mix containing 1 U of T4 DNA ligase (2 U/μl; Fermentas UAB, Vilnius, Lithuania), 1× ligation buffer (40 mM Tris-HCl pH 7.8, 10 mM MgCl_2_, 10 mM DTT, 0.5 mM ATP; Fermentas UAB, Vilnius, Lithuania), and 1 pmol adapter (adXbaI) in each of 5 μl volumes. 5 μl of the ligation mixture was added to 20 μl of digested DNAs and the tubes were incubated for 30 min at 18–22 °C, and then to inactivate the T4 DNA ligase for 10 min at 70 °C.

### pehX-LM PCR/XbaI amplification

PCRs were performed in 25-μl volumes using 10 pmol of the *pehX*-specific primers (pehX1 and pehX8), 5 pmol of adapter-specific primer (p-adXbaI; 5′-CTCACTCTCACCAACGTCGACCTAGA-3′), 2.5 μl of a deoxynucleoside triphosphate mixture (2 mM concentration of each), 1 U of *Taq* polymerase (1 U/μl, Fermentas UAB, Vilnius, Lithuania), 2.5 μl of 10× PCR buffer *Taq* with (NH_4_)_2_SO_4_ (Fermentas UAB, Vilnius, Lithuania), 2 μl of 20 mM MgCl_2_, and 1 μl of adapter-ligated DNA fragments. The PCR was performed as follows: 5 min at 94 °C to release an unligated helper oligonucleotide, 5 min at 72 °C to fill in the single-stranded ends of the adapter and create amplicons, 25 cycles of denaturation at 94 °C for 30 s, 30 s of annealing at 60 °C and an extension step at 72 °C for 180 s. After the last cycle, the samples were incubated at 72 °C for 10 min.

### Test to verify the specificity of the amplification in the pehX-LM PCR/XbaI method

Two extra PCR samples were prepared for each of the *K. oxytoca* reference strains and for 50 randomly selected clinical strains: one sample contained only a pair of the *peh*X-specific primers (pehX1 and pehX8) and the other contained only an adapter-specific primer (p-adXbaI). The PCR conditions for *pehX*-LM PCR/XbaI amplification were as described above. To check that there is no specific *pehX*-PCR product in the *pehX*-LM PCR/XbaI analysis, we tested 53 strains of other species of *Enterobacteriaceae* family (including 25 clinical and 3 reference *Klebsiella pneumoniae* strains (ATCC 25926; ATCC 700603™; ATCC® 13884™) 10 clinical strains of *E. coli*, 10 of *S. marcescens*, 5 of *P. vulgaris*) using the same protocol as for *K. oxytoca*.

### PCR MP genotyping

All *K. oxytoca* were genotyped by PCR MP according to the procedure described by Stojowska et al. (2009a).

### Detection and fingerprint pattern analysis

We analysed 10 μl out of 25 μl of a PCR mixture by electrophoresis (2 % agarose gels consisting of 0.5 μg/ml of ethidium bromide, 100 V, for 1 h in 1× TAE buffer). We studied and archived gel images using Versa Doc 1000 Imaging System (Bio-Rad Laboratories, Hercules, USA). The patterns we obtained from the electropherograms were converted and analysed using the Quantity One software package, version 4.3.1 (Bio-Rad, San Francisco, CA, USA). Band positions in each gel were normalised using the DNA ladder (the molecular DNA size marker: 100–1000 bp, BLIRT SA, DNA Gdańsk, POLAND). Band matching and isolate similarity were accomplished using the Dice band-based coefficient of similarity. Tolerance and optimisations settings were 2.0 % for both *pehX*-LM PCR/XbaI and PCR MP. A dendrogram was constructed using the Unweighted Pair Group Method with Arithmetic Mean (UPGMA).

### Reproducibility of the pehX-LM PCR/XbaI method

To check the reproducibility of the *pehX*-LM PCR/XbaI method, ten randomly selected strains were genotyped by this method in three independent experiments performed with DNA isolated from different cultures of one strain. The percentage similarity among fingerprint patterns for each gel was recorded and the average across the gels was calculated. Moreover, two different thermal cyclers (a Biometra Tgradient cycler and an Eppendorf MasterCycler EP gradient) and two different *Taq* polymerases (Fermentas UAB, Vilnius, Lithuania and BLIRT SA, DNA Gdańsk, POLAND) were used by two independent persons.

### Method sensitivity-detection limit

A single colony of bacteria is sufficient for DNA isolation. 10–25 ng of genomic DNA is required in a single reaction to obtain DNA patterns.

## Results

### Outline of the pehX-LM PCR/XbaI method

**Fig. 1:**
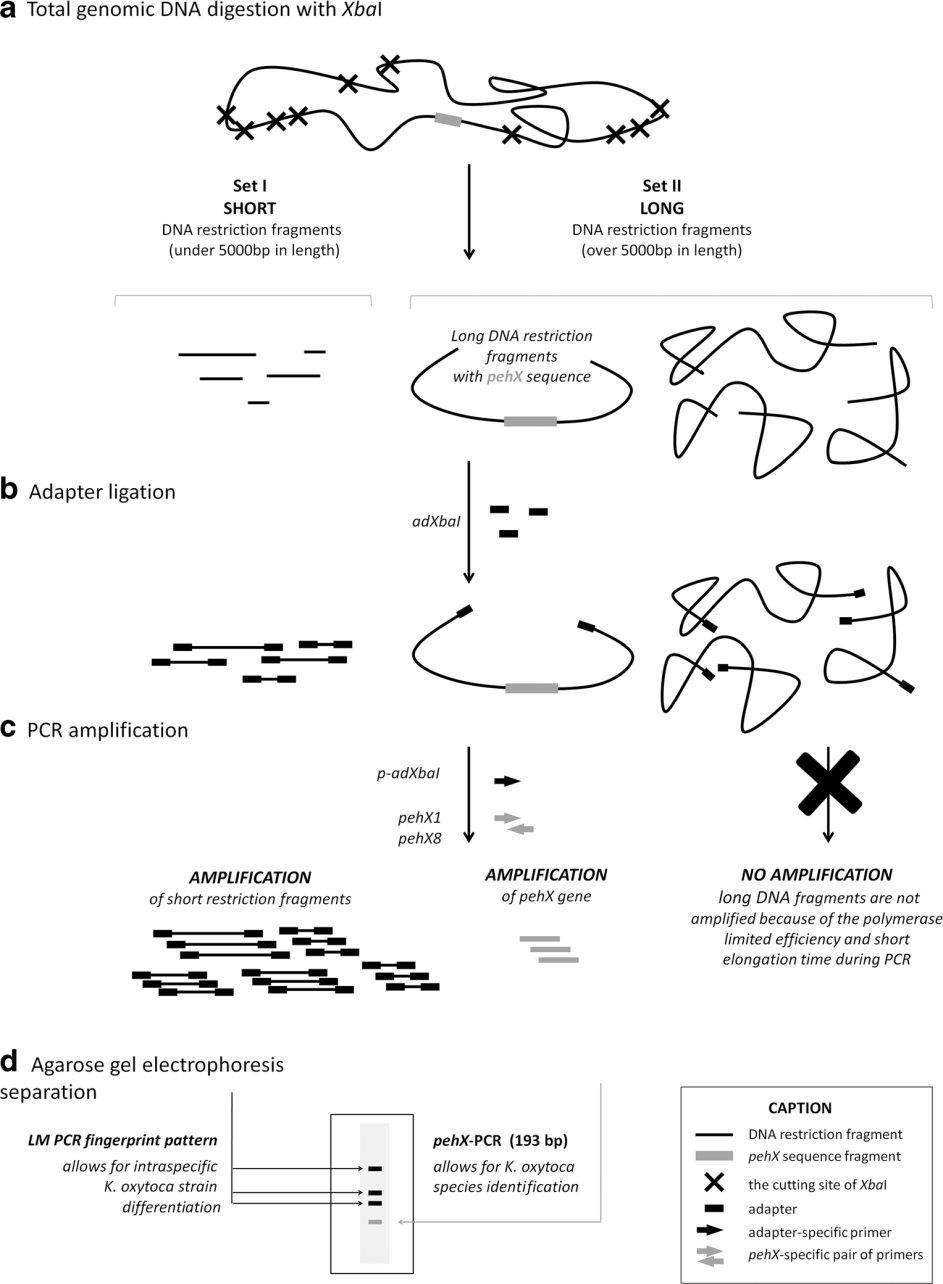
Outline of *pehX*-LM PCR/XbaI method. I, II sets of restriction fragments. Caption section (*bottom right corner*) shows the description of all symbols used in the *pehX*-LM PCR method scheme.

**Fig. 2:**
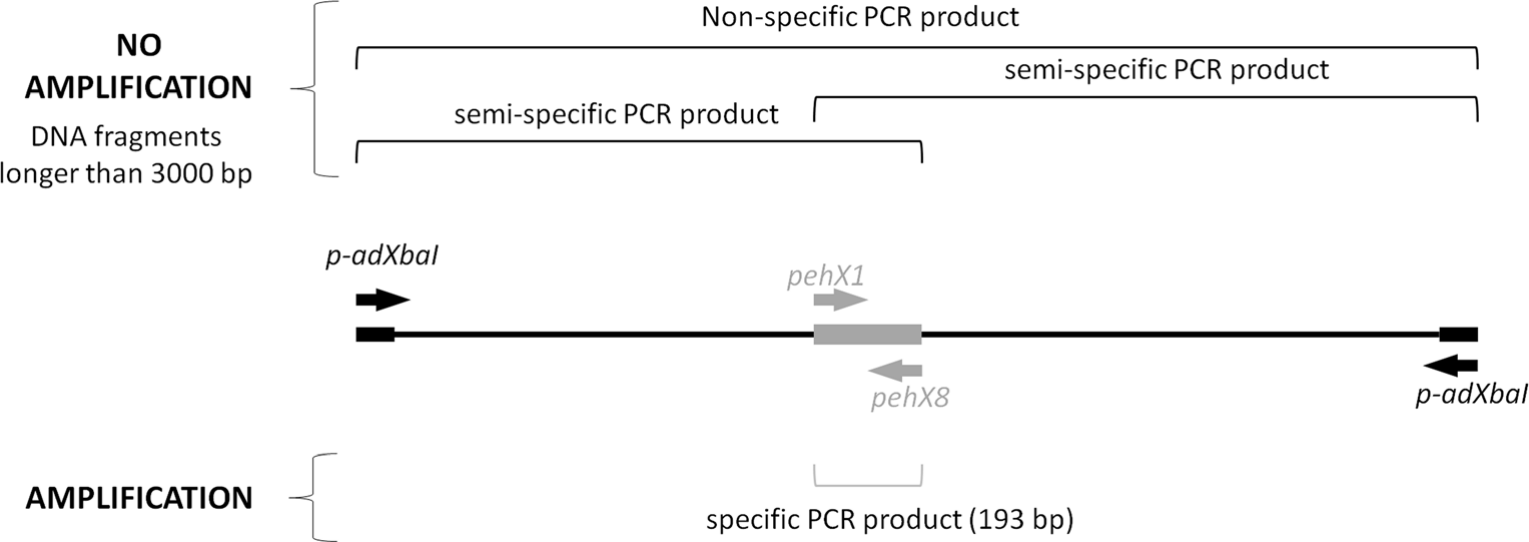
Specific amplification of *pehX* gene from *Xba*I restriction fragment. p-adXbaI—adapter-specific primer, pehX1 and pehX8—*pehX-*specific primers. The *pehX* gene is located amongst long restriction fragments, more than 3000 bp away from each end

To perform the species-specific fingerprinting of *K. oxytoca* strains, we applied the LM PCR technique involving the ligation and selective amplification of DNA restriction fragments in combination with the specific gene amplification of *pehX*-genes. The method used in our experiments is shown in Fig. 1. A genomic total DNA is digested completely with the restriction enzyme (XbaI) (Fig. 1a). We chose XbaI because it cuts the genomic DNA of *Enterobacteriaceae* bacteria at no more than 40 restriction sites and generates up to 10 fragments from 300 to 3000 bp in length (Fig. 1a, set I, SHORT fragments). The remaining fragments are usually much longer than 5000 bp (Fig. 1a, set II, LONG fragments) and one of them contains the *pehX* gene. The *pehX* gene is located at a distance of over 3000 bp from each end of the restriction fragment which avoids a semi-specific amplification (Fig. 2). All the restriction fragments contain 5′ cohesive ends and are ligated to the synthetic adapter (adXbaI) in the next step (Fig. 1b). At first, the unligated oligonucleotides (aXbaHELP) are released and single-stranded ends are filled with DNA polymerase. Following this, DNA fragments are amplified by PCR using the adapter-specific primer (p-adXbaI) capable of hybridising with the 5′ ends of DNA fragments. Only SHORT DNA fragments are amplified (Fig. 1, set I) and after separation by agarose gel electrophoresis they form strain-specific band patterns (Fig.1d). LONG DNA fragments are not amplified because the DNA polymerase has a limited efficiency and a short elongation time (Fig. 1, set II). Addition of *pehX*-specific primers during the PCR amplification process (Fig. 1c) creates an extra PCR product with a length of 193-bp, an indication that *K. oxytoca* species are present (Fig. 1d). Three primers should be used in one PCR to receive a full *K. oxytoca* strain fingerprint profile: the adapter-specific primer (p-adXbaI) to amplify strain-specific restriction fragments and a pair of *pehX*-specific primers (pehX1 and pehX8) to amplify species-specific DNA fragments.

### Verification of the pehX-LM PCR/XbaI method

**Fig. 3:**
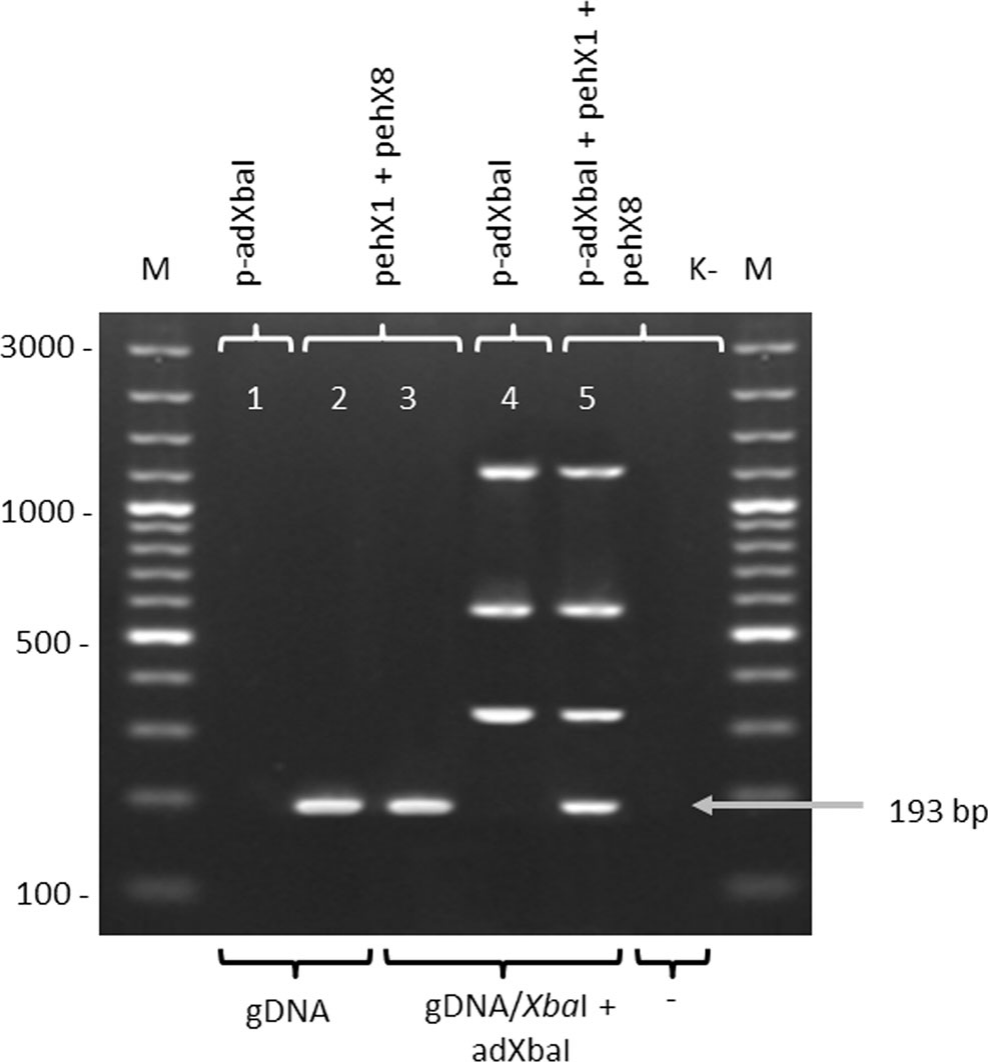
Verification of the *pehX*-LM PCR/XbaI model system for the *K. oxytoca* reference strain. Electropherogram of PCR products which were obtained using a combination of *pehX*-specific or adapter-specific primers with digested or undigested DNA. gDNA—non digested, genomic DNA of *K. oxytoca* K26, gDNA/XbaI + adXbaI—digested and ligated to the adapter DNA of *K. oxytoca* K26; p-adXbaI—adapter-specific primer, pehX1 and pehX8—pehX-specific primers; M—DNA ladder (100–3000 bp), K-negative control (without DNA)

The *pehX*-LM PCR/XbaI method was verified. The results are shown in Fig. 3. The *pehX*-species-specific fragments can be amplified by PCR using the pehX1 and pehX8 primers either from undigested genomic DNA (Fig. 3, lane 2) or the DNA previously digested and ligated to form the adapter (Fig. 3, lane 3), whilst the strain-specific fingerprint profile (LM PCR) can be obtained only after the complete digestion of the genomic DNA, the ligation of the adapter to the fragments and PCR amplification with the use of the p-adXbaI primer (Fig. 3, lanes 1 and 4). The complete fingerprint profile of the *K. oxytoca* strain consists both of a *pehX*-specific product with a length of 193 bp and three well separated strain-specific LM PCR products with a length of at least 300 bp (Fig. 3, lane 5). There are no extra non-specific or semi-specific PCR products with three primers in one PCR.

**Fig. 4:**
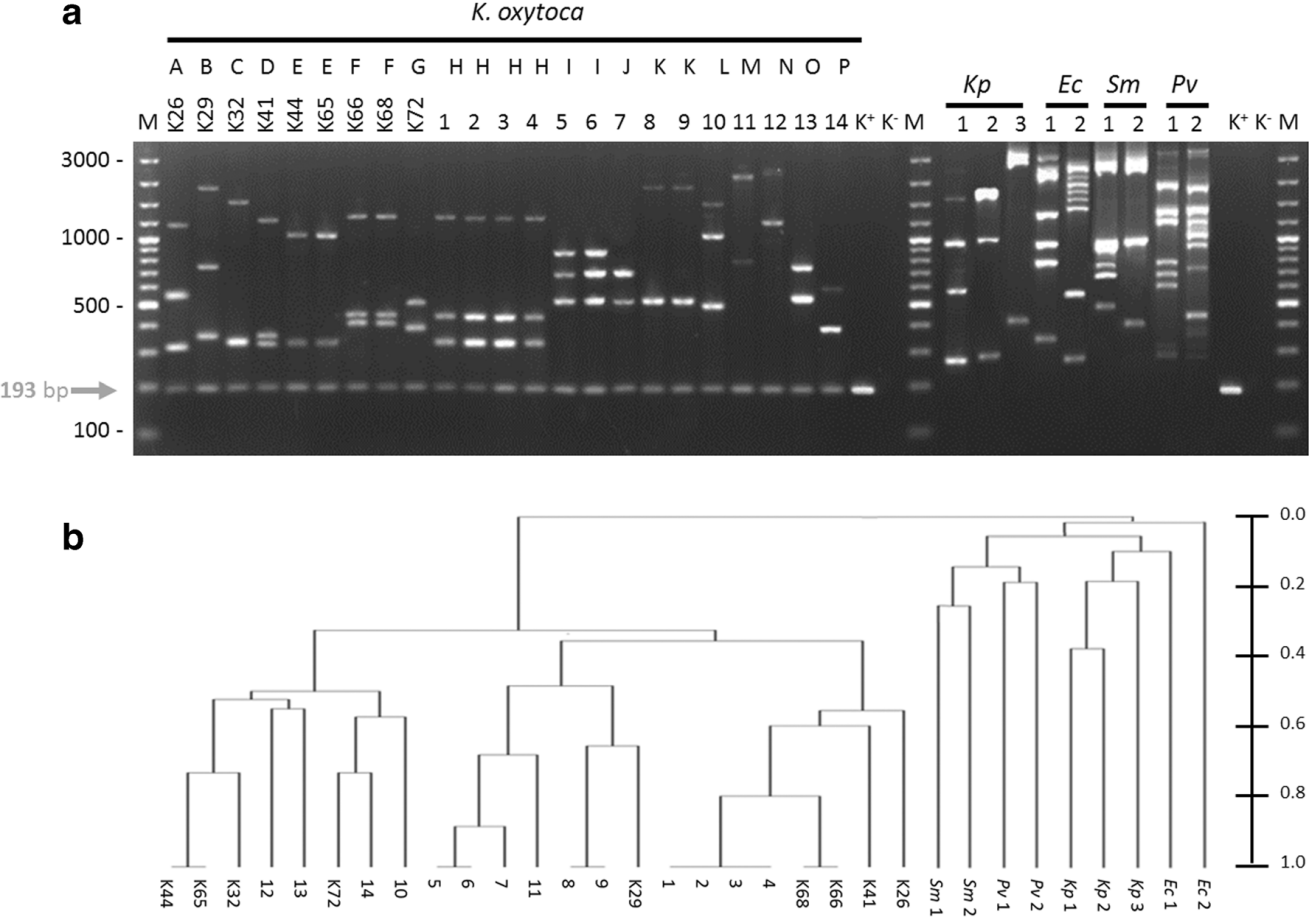
**a** Representative typing results for *Enterobacteriaceae* by *pehX*-LM PCR/XbaI method. **b** Dendrogram of *pehX*-LM PCR/XbaI constructed under Dice band-based coefficient of similarity and the Unweighted Pair Group Method with Arithmetic Mean (UPGMA). K26-K72 reference strains of *K. oxytoca* from Ørskov collection, 1-14 - clonally related and unrelated *K. oxytoca* strains, *Kp - Klebsiella pneumoniae*, *Ec - Escherichia coli*, *Sm - Serratia marcescens*, *Pv - Proteus vulgaris*, A-P genotypes (different fingerprint profiles), K+ positive control of *pehX*-specific product (193-bp DNA fragment amplified from genomic DNA of *K.* *oxytoca* K26). K- negative control of PCR (without DNA), M - DNA ladder (100–3000 bp)

To demonstrate the usefulness and discrimination power of the *pehX*-LM PCR/XbaI method, we examined 200 clinical and 9 references *K. oxytoca*, 28 *K. pneumoniae* and 25 strains of other species of *Enterobacteriaceae*. The typing results for selected strains are shown in Fig. 4a.

For all *K. oxytoca* strains the electrophoretic profile usually contained 2, 3 or 4 strain-specific (genotype-specific) LM PCR products with a length of at least 300 bp, and one well-separated *pehX*-specific product with a length of 193 bp. The electrophoretic patterns relating to *K. pneumoniae* and the other bacteria from *Enterobacteriaceae*, contained up to 10 well visible and well-separated genotype-specific LM PCR products. There was no *pehX*-specific band in the profiles. The presence of a *pehX*-specific product in all *K. oxytoca* strains and its absence in other *Enterobacteriaceae* strains in the electrophoretic profile was confirmed by comparing the results of three experiments with reference strains as shown in Fig. 3, lines 3, 4 and 5. All *K. oxytoca* strains tested positive on PCR with the primer pair pehX1/pehX8, whereas the negative results were obtained for all *K. pneumoniae* (28 strains) and *S. marcescens* (10 strains), *E. coli* (15 strains), *P. vulgaris* (10 strains). Furthermore, there were no semi-specific PCR products generated by pehX1/p-adXbaI or/and pehX8/p-adXbaI primers in both *K. oxytoca* strains and non-*K. oxytoca* strains.

The typeability, reproducibility and discriminatory power of *pehX*-LM PCR/XbaI technique were evaluated. The typeability of the *pehX*-LM PCR/XbaI method was at 100 % (for each *K. oxytoca* strain electrophoretic pattern with species-specific and genotype-specific bands was obtained), whilst reproducibility was estimated at 96,8 %. We have proved that genotyping results do not depend on the thermostable DNA polymerase in PCR and the thermal cycler used (Fig. S1). The same results were obtained by two independent researchers giving the same genetic groups.

The new *pehX*-LM PCR/XbaI method was shown to have a high discriminatory power. Most of 209 *K. oxytoca* strains showed a single-specific pattern. We distinguished 187 different genotypes at a cut-off similarity level of 95 %, which conforms to the PCR MP results (cut-off values 90 %) (Stojowska et al. 2009b). The genotyping results for selected *K. oxytoca* by the *pehX*-LM PCR/XbaI method and the PCR MP method are shown in Fig. 4a and Fig. S2, respectively. Typing by *pehX*-LM PCR/XbaI lead to the same electrophoretic patterns for clonally related strains but clonally unrelated strains generated different electrophoretic profiles.

All the patterns for *K. oxytoca* were different from those observed for *K. pneumoniae* and other *Enterobacteriaceae* (Fig. 4a). Measured with the use of the Dice band-based coefficients the UPGMA method, *K. oxytoca* strains had a similarity of over 30 %, whilst similarity between *K. oxytoca* and other *Enterobacteriaceae* strains did not exceed 5 % (Fig. 4b).

## Discussion

Currently, the complete identification of *Klebsiella* spp. and the reliable strain typing analysis can only be obtained with the use of separate molecular tests. A number of different molecular techniques have been developed for genetic typing including ribotyping (ITS PCR) (Stojowska et al. 2009b), pulsed field gel electrophoresis (PFGE, using *Xba*I restriction enzyme) (Fujita et al. 2015; Izdebski et al. 2015; Krawczyk et al. 2005), multilocus sequence typing (MLST, based on *ropB*, *gapA*, *mdh*, *pgi*, *phoE* and *tonB* genes) (Fujita et al. 2015; Izdebski et al. 2015), DNA fingerprinting based on PCR such as random amplified polymorphic DNA (RAPD) (Brisse and Verhoef 2001; Krawczyk et al. 2005; Vogel et al. 1999), AFLP (using restriction enzymes *Eco*RI + *Mse*I) (Donnarumma et al. 2012; Jonas et al. 2004; van der Zee et al. 2003), Amplification of DNA fragments Surrounding Rare Restriction Sites (ADSRRS) (Krawczyk et.al. 2005), and PCR MP (using restriction enzyme *Hind*III) (Stojowska et al. 2009a, b). None of these methods will not provide information about the species or genus of the tested strains. For this reason, they require the use of purified DNA from the correctly identified species of bacteria. Closely related species which belong to the same genus may hinder the diagnosis.

In this study, we propose a new simple method called *pehX*-LM PCR/XbaI, which uses fingerprint patterns to show whether the tested strain belongs to the *K. oxytoca,* and identify its genotype. *pehX*-LM PCR/XbaI is a combination of the following two methods: the species-specific amplification of the *pehX* gene and the non-specific amplification of short restriction fragments (LM PCR). The only way to make this combination work was to select suitable restriction enzyme *Xba*I, which (i) rarely cuts genomic DNA of *Enterobacteriaceae*, (ii) generates a small number of relatively short restriction fragments (up to 3000–5000 bp) and may be amplified using the standard DNA polymerases and (iii) is able to detect a strain-specific restriction fragment length polymorphism using gel electrophoresis. Lack of amplification of long, non-specific DNA restriction fragments and both semi-specific fragments generated with the adapter-specific and *pehX*-specific primers can be attributed to the limited efficiency of the DNA polymerase and short elongation times during the PCR cycles.

As with any other LM PCR method, the *pehX*-LM PCR/XbaI requires the use of a purified genomic DNA from one species/strain of bacteria (typically, DNA isolated from one colony of bacteria). If a sample contains a mix of bacteria, the fingerprint patterns will be distorted. If this happens, the number of bands in the profile will increase (more than four bands) and the bands will not be separated well after gel electrophoresis. When there is no *K. oxytoca* DNA in a sample (a mix or a single strain), there will be no amplification of a specific fragment of the *pehX* gene (193 bp). The length of PCR products in the fingerprint pattern will not be less than 300 bp. The *pehX*-LM PCR/XbaI method may help identify *K. oxytoca* in a sample and determine the purity of the sample (a single strain or a mix of bacteria), which is impossible using other LM PCR methods.

The new *pehX*-LM PCR/XbaI method has a high discriminatory power, comparable to other LM PCR methods. Fingerprint patterns consist of up to five bands and they are easy to read and interpret. Simultaneous species identification allows avoiding mistaken identities or contamination which could lead to the misinterpretation of fingerprint patterns. Analyses (without DNA isolation) take no more than 4 h. This is of extreme importance, especially in hospital settings where time may play a crucial role in diagnosis and treatment. The *pehX*-LM PCR/XbaI method does not require a high degree of technical competency. To be reproducible, analyses do not require careful standardization of the PCR conditions and are not sensitive to small temperature fluctuations during PCR.

The rarely cutting restriction enzymes, e.g., *Xba*I (as in *pehX*-LM PCR/XbaI method) give a small number of the short restriction fragments and the number and the size (length) of them depend on the genetic events (deletion, insertion, mutation). Our studies have indicated that this is sufficient for differentiation of *K. oxytoca strains.*

However, we cannot exclude that the clonally unrelated strains will obtain the same electrophoretic pattern and will be classified as the same genotype. It is a disadvantage of all typing methods that use the restriction enzymes for whole genome digestion. It is recommended to use another method for confirmation (e.g., identification of nosocomial infection) for doubtful results.

We cannot ensure that for non-*K. oxytoca* strains belonging to another *Enterobacteriaceae*; there will be an unspecific 190–200 bp band in the fingerprint pattern, and that it may hinder the proper identification. In this case *K. oxytoca*-species identification requires confirmation. It is recommended to repeat the PCR without pehX-specific primers (as it was shown on Fig. 3, lane 4). At the time the species-specific fragment for *K. oxytoca* should not be observed.

A small number of strain-specific PCR products make fingerprint patterns well readable.

It enables to generate a dendrogram graph to show genetic diversity between tested strains but it could be insufficient to measure the level of genetic similarity, to study bacterial population dynamic or transmission and sources of bacterial infections (more advanced clinical study). This method allows to distinguish clonally related strains from clonally unrelated strains. It may be used as a screening method for the initial identification of nosocomial infection in hospitals.

The *pehX*-LM PCR/XbaI method was designed for *K. oxytoca* typing. Like with other LM PCR methods, it is not universal and cannot be applied for other species. By changing the species-specific primers (specific DNA target) and/or restriction enzymes in the *pehX*-LM PCR/XbaI method, it is possible to design similar assays for other species or genera of bacteria.

To sum up:

1. The *pehX*-LM PCR/XbaI method was designed to simultaneously type *K. oxytoca* strains and confirm the species.
2. The method combines the merits of species-specific PCR primers, carefully selecting sequences which are present only in *K. oxytoca*, enabling a quick confirmation of the species with the use of the LM PCR method for the genotyping of strains.
3. Unlike in most fingerprinting methods, we obtain a clear pattern (approx. 3–5 bands) with a sufficient, relatively high discriminatory power.
4. A small amount of DNA is used as a template for PCR. This reduces the duration and cost of a single analysis.
5. The method after validation process could be used in clinical and environmental research.

## Electronic supplementary material

ESM 1 (PDF 354 kb)
