# Supplementary material for: A new assay for the simultaneous identification and differentiation of Klebsiella oxytoca strains
Source: Appl Microbiol Biotechnol. 2016 Oct 8;100(23):10115–23. doi: 10.1007/s00253-016-7881-1 (PMC5102950; doi:10.1007/s00253-016-7881-1)
Supplement: Supplementary file 1 — (PDF 354 kb) [file 253_2016_7881_MOESM1_ESM.pdf]

Supplementary Material

Applied Microbiology and Biotechnology

A new assay for the simultaneous identification and differentiation of *Klebsiella oxytoca* strains

Karolina Stojowska-Swędrzyńska, Beata Krawczyk*

Gdańsk University of Technology, Department of Molecular Biotechnology and Microbiology, ul. G. Narutowicza 11/12, 80-233 Gdańsk, POLAND

*Corresponding author: Beata Krawczyk, Gdańsk University of Technology, Department of Molecular Biotechnology and Microbiology, ul. G. Narutowicza 11/12, 80-233 Gdańsk, POLAND; Phone:+48 58347 23 83; Fax:+48 58347 18 22

Email: beakrawc@pg.gda.pl

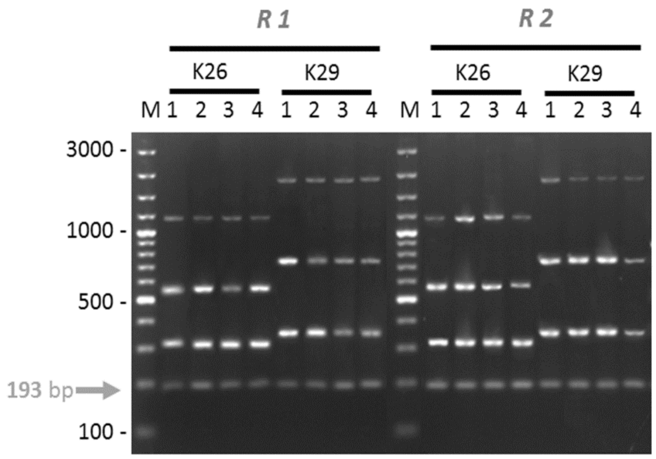

Fig. S1. Results of the reproducibility test. R1, R2 - researchers in two independent experiments; K26 and K29 – *K. oxytoca* tested strains; 1, 2 - the result of PCR with *Taq* polymerase, Fermentas UAB, Vilnius, Lithuania and with *Taq* polymerase, BLIRT SA, Gdańsk, POLAND respectively and Eppendorf MasterCycler EP gradient thermocycler; 3, 4 – the result of PCR with *Taq* polymerase, Fermentas UAB, Vilnius, Lithuania and with *Taq* polymerase, BLIRT SA, Gdańsk, POLAND respectively and Biometra Tgradient thermocycler; M – DNA ladder (100 – 3000 bp).

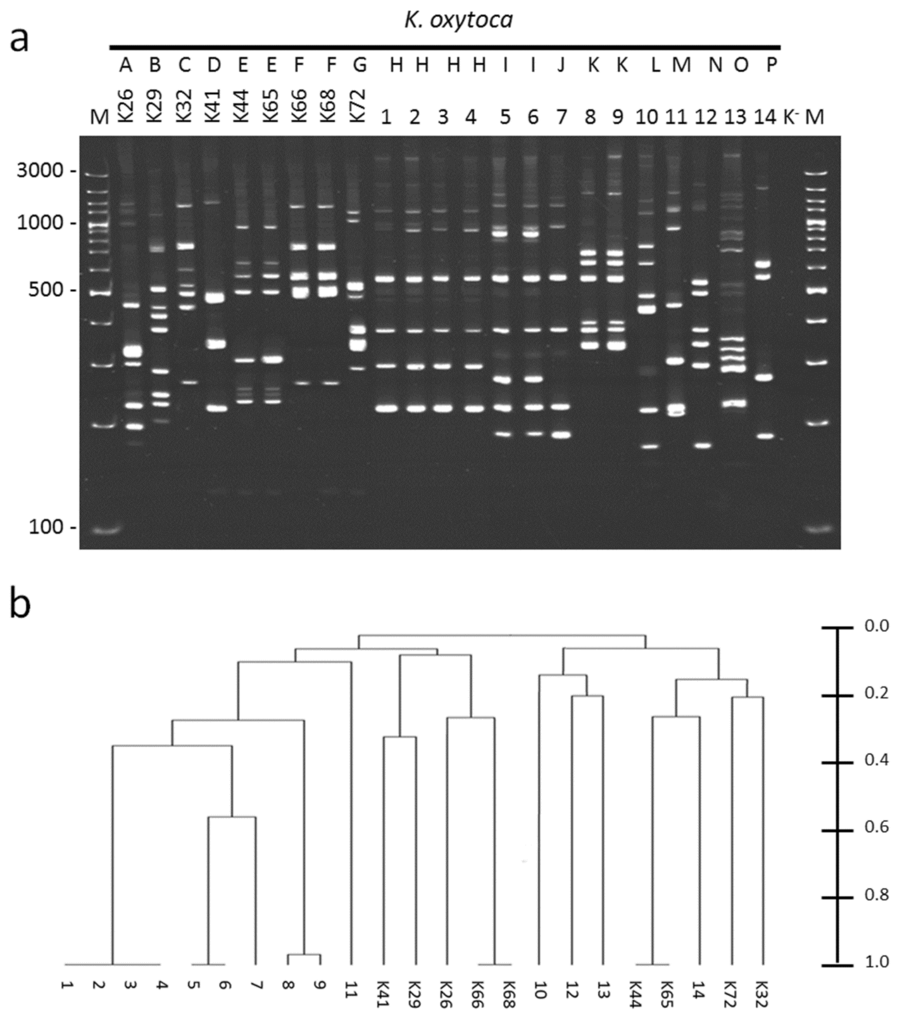

Fig. S2 (a) Representative typing results for *K. oxytoca* strains by PCR MP method, (b) dendrogram of PCR MP constructed under Dice band-based coefficient of similarity and the Unweighted Pair Group Method with Arithmetic Mean (UPGMA). K26- K72 reference strains of *K. oxytoca* from Ørskov collection, 1-14 – clonally related and unrelated *K. oxytoca* strains, A-P – genotypes , Kp1 - *K. pneumoniae*. K- negative control of PCR (without DNA), M – DNA ladder (100 – 3000 bp).
